# AI‐Equipped Scanning Probe Microscopy for Autonomous Site‐Specific Atomic‐Level Characterization at Room Temperature

**DOI:** 10.1002/smtd.202400813

**Published:** 2024-09-06

**Authors:** Zhuo Diao, Keiichi Ueda, Linfeng Hou, Fengxuan Li, Hayato Yamashita, Masayuki Abe

**Affiliations:** ^1^ Graduate School of Engineering Science Osaka University 1‐3 Machikaneyama‐Cho Toyonaka Osaka 560‐8531 Japan; ^2^ Tokyo Metropolitan Industrial Technology Research Institute 2‐4‐10 Aomi Koto‐Ku Tokyo 135‐0064 Japan

**Keywords:** deep learning, room temperature, scanning probe microscopy, scanning tunneling spectroscopy, self‐driving

## Abstract

An advanced scanning probe microscopy system enhanced with artificial intelligence (AI‐SPM) designed for self‐driving atomic‐scale measurements is presented. This system expertly identifies and manipulates atomic positions with high precision, autonomously performing tasks such as spectroscopic data acquisition and atomic adjustment. An outstanding feature of AI‐SPM is its ability to detect and adapt to surface defects, targeting or avoiding them as necessary. It is also designed to overcome typical challenges such as positional drift and tip apex atomic variations due to the thermal effects, ensuring accurate, site‐specific surface analysis. The tests under the demanding conditions of room temperature have demonstrated the robustness of the system, successfully navigating thermal drift and tip fluctuations. During these tests on the Si(111)‐(7 × 7) surface, AI‐SPM autonomously identified defect‐free regions and performed a large number of current–voltage spectroscopy measurements at different adatom sites, while autonomously compensating for thermal drift and monitoring probe health. These experiments produce extensive data sets that are critical for reliable materials characterization and demonstrate the potential of AI‐SPM to significantly improve data acquisition. The integration of AI into SPM technologies represents a step toward more effective, precise and reliable atomic‐level surface analysis, revolutionizing materials characterization methods.

## Graphical abstract

The advanced AI‐enhanced scanning probe microscopy (AI‐SPM) system autonomously performs atomic‐scale measurements at room temperature, identifying and manipulating atomic positions with high precision. It adapts to surface defects, compensates for positional drift and thermal effects, and ensures accurate surface analysis. Tests on the Si(111)‐(7 × 7) surface confirmed its robustness and provided comprehensive, reliable data for materials characterization, demonstrating the potential of AI‐SPM to revolutionize atomic‐level analysis.

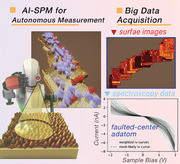

## 1 Introduction

The integration of artificial intelligence (AI) with nanotechnology has been recognized as critical since the pioneering work by Drexler in 1986.^[^ ^1^ ^]^ However, the advances described in that book have not been widely realized. As a potential technique to reveal the advances described in that book, scanning probe microscopy (SPM) has emerged in the characterization of nanoscale surfaces, enabling the discovery of new surface properties and phenomena.^[^ ^2^, ^3^ ^]^ The upcoming “The 25th International Conference on Non‐Contact Atomic Force Microscopy” (NC‐AFM 2024) will even feature a satellite workshop on AI and machine learning techniques in atomic force microscopy (AFM),^[^ ^4^ ^]^ highlighting the growing importance of AI‐based SPM (AI‐SPM) in advancing its capabilities in material science. Today, SPM has made significant contributions to both basic science and industrial applications. In particular, in the field of basic science, it can not only image surfaces but also measure the physical properties of individual atoms and move atoms to create structures.^[^ ^5^ ^]^ Most of these experiments have been performed in cryogenic environments. This is because not only the SPM instrument but also the apex of the SPM tip and the sample itself are thermally stable. However, from a practical standpoint, conducting these processes at room temperature is essential.

Even in room temperature environments, where thermal effects can affect measurements, SPM has provided significant capabilities such as dynamic imaging to observe temporal changes in chemical reactions,^[^ ^6^, ^7^ ^]^ biological processes,^[^ ^8^ ^]^ surface dynamics,^[^ ^9^, ^10^ ^]^ diffusion,^[^ ^11^, ^12^ ^]^ and crystal growth.^[^ ^13^ ^]^ In addition, there are studies of dopant atom manipulation that can be done at room temperature.^[^ ^14^, ^15^ ^]^

There have been challenges in further pursuing these pioneering room‐temperature experiments. Using the room‐temperature SPM to achieve atomic resolution does not eliminate the thermal effects on the instrument which leads to measurement instability. One of the most significant effects of fluctuations in atomic resolution SPM measurements is thermal drift and piezo creep. These phenomena continuously change the relative position of the tip and sample atoms, which not only prevents continuous measurement of the same area but can also cause image distortion. Another problem of the room temperature SPM is the frequent change of the tip apex. The quality of the image is affected by the frequent change of the tip apex atom especially in atomic resolution imaging.^[^ ^16^, ^17^, ^18^ ^]^ Repairing the tip apex is usually done by touching to the surface, which requires time and a great deal of attention. To achieve high‐precision measurements under such inherently non‐optimized conditions, SPM techniques such as drift correction^[^ ^19^, ^20^ ^]^ and tip fabrication^[^ ^17^, ^21^ ^]^ become indispensable in the context of room temperature SPM. However, even with these techniques, it has been difficult to perform site‐specific experiments at the atomic level at room temperature.

In recent years, to overcome the limits of human capability, experiments utilizing the concept of the self‐driving laboratory^[^ ^22^, ^23^ ^]^ have become increasingly prevalent, driven by the demand for extensive and complex data collection. This approach has risen to prominence as a key solution for challenges like new materials discovery, largely through the application of AI. The self‐driving concept is also anticipated to be highly beneficial for time‐consuming and labor‐intensive SPM experiments, which are manually operated.^[^ ^24^, ^25^ ^]^ A deep learning model, adept in signal processing and computer vision, can identify specific patterns with accuracy nearly on par with human experts.^[^ ^26^, ^27^ ^]^ Strategically integrating AI can significantly reduce the dependency on manual intervention in SPM operations.^[^ ^28^ ^]^

There have been previous studies on the integration of image recognition AI and SPM that have made notable contributions to the fields of data analysis and processing.^[^ ^29^, ^30^, ^31^, ^32^, ^33^ ^]^ The application of these AI‐driven analysis techniques to the processing of real‐time measured data has the potential to significantly automate SPM operations. The effective use of such technologies requires a complex integration into the control mechanisms, both hardware and software, including scanning protocols and AI functions, as well as the acquisition of extensive data sets for the cultivation of highly efficient AI models. Particularly in cryogenic conditions, where thermal perturbations are greatly reduced, recent research has underscored the utility of AI‐assisted methods in promoting autonomous scanning^[^ ^34^, ^35^, ^36^ ^]^ and single atom manipulation.^[^ ^37^ ^]^ Comparatively, room temperature environments require more sophisticated AI to automate SPM measurement tasks than cryogenic ones. This enhancement is necessary to compensate for the challenges posed by thermal fluctuations and to ensure the reproducibility of experiments. Robustness in handling measurements under unstable conditions is critical for performing real‐time and site‐specific experiments at room temperature. In addition, it is important to recognize during data collection that the simulated data sets used to train^[^ ^30^, ^32^ ^]^ may not fully capture the variations in atomic images. This limitation is due to differences in tip state and adsorbate patterns in actual measurements. Addressing these considerations is critical to improving the reliability and performance of site‐specific SPM measurements at room temperature.

In this manuscript, we present a deep learning‐based AI‐SPM system specifically designed for site‐specific operation at room temperature. Our AI‐SPM system is an integrated fusion of software, control firmware, and hardware components that facilitates the development of a robust neural network through a systematic data collection process. Decision making and optimization, tailored to room temperature conditions, are fully automated to achieve atomic precision measurements. Using this system, we demonstrate two key applications in surface characterization: autonomous acquisition of high quality images and large data sets for atomic precision scanning tunneling spectroscopy (STS) at room temperature.

## 2 AI‐SPM for Self‐Driving Measurement

### 2.1 AI‐SPM Configuration

**Figure 1:**
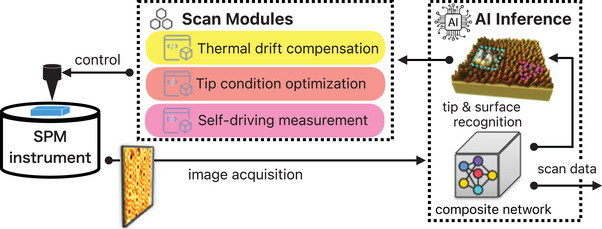
Schematic of the Artificial Intelligence Scanning Probe Microscopy (AI‐SPM) system. The system compensates for thermal drift and unfavorable tip state, which is an inherent problem at room temperature. It then determines the presence of adsorption sites and defects, and performs site‐specific experiments at targeted atomic positions. Large amounts of data can be acquired automatically.

**Figure** **1** shows the configuration of our AI‐SPM system. The SPM hardware is conventional, and two main program parts are added to our home‐built scan software for AI operation. The AI inference section receives the SPM measurement data, makes a situational judgment, and determines the next task. In particular, it uses the images to determine the state of the tip apex, the identification of individual atomic sites and unit cells, the location and type of adsorbates, and whether further site‐specific measurements are possible. The tip and sample surface information sent by the AI inference component to the Scan Module part is used in the operator‐programmed experiment. In Figure 1, the Scan Module includes two scripts essential for the room temperature experiment (thermal drift compensation and tip condition optimization), as well as a self‐driving measurement script. These scripts are used in the experiments in this manuscript.

### 2.2 Convolutional Neural Networks on AI Inference

The acquired image data of the surface not only confirms the crystal structure of the surface but also provides information on the condition for site‐specific measurement: the presence of defects or adsorbates, not atomically clean area or steps, tip apex condition. To automatically make decisions on all this information, we have used convolutional neural networks (CNNs). Each CNN is tailored for a specific prediction task, and they all use scanned topography as input. By integrating these CNNs into a composite network, we provide comprehensive access to a wide range of information about the real‐time scanning topography.^[^ ^38^ ^]^ Understanding both tip and surface conditions enables site‐specific measurements such as STS and atomic manipulation.

**Figure 2:**
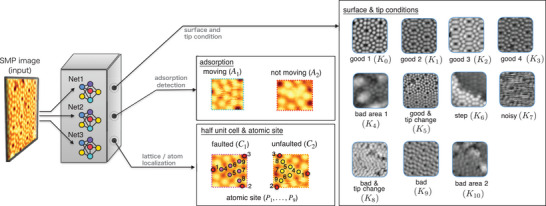
Convolutional Neural Networks (CNNs Net1, Net2, and Net3) are used in this study for room temperature site‐specific analysis of the Si(111)‐(7 × 7) surface. Net1 is tasked with detecting tip and sample conditions, Net2 identifies adsorption sites, and Net3 is responsible for detecting half‐unit cells and atomic positions.

**Figure** **2** shows the CNN architecture for the Si(111)‐(7 × 7) surface. We have designed a composite network structure consisting of three different models, Net1, Net2, and Net3, each dedicated to a specific task. Net1 is tasked with recognizing the conditions of both the tip and the sample by performing a multi‐class classification of tip apex and surface conditions *K*_*i*_(*i* = 0, 1, ⋅⋅⋅, 10).^[^ ^38^ ^]^ It determines whether the sample and the tip are both in a state (*K* = 1, 2, 3, 4) that allows for site‐specific measurements. If the surface is not contaminated and the tip is capable of atomic resolution, it is considered to be in a “good” state for site‐specific measurement. We have found that Net1 has an accuracy of 87–90% (see Figure S1a Supporting Information). The reason for classifying good into four categories is to determine and create a tip apex that can be used for atomic manipulation in the future.^[^ ^39^, ^40^ ^]^

Net2 determines adsorbates. On the Si(111)‐(7 × 7) we can find some adsorbates at the atomic level that are moving and some that are not. Here we define the immobile adsorbate *A*_2_ as the one that is imaged brighter than the Si adsorbate in the half‐unit cell. Another type of adsorbate is those that are imaged like noise or feedback errors. These are adsorbates in motion that remain in a half‐unit cell (*A*_1_ in Figure 2). The moving adsorbates in the half‐unit cell have been studied for clustering and atomic manipulation experiments.^[^ ^41^, ^42^, ^43^, ^44^, ^45^, ^46^, ^47^, ^48^ ^]^ Net2 is designed to be applied to these studies as well.

Net3 detects site‐specific information of the Si(111)‐(7 × 7) surface. Half‐unit cells are categorized as bounding boxes of *C*_1_ and *C*_2_, respectively. By applying a negative sample bias during the measurement and observing the response of the screen, it is possible to determine whether *C*_1_ or *C*_2_ corresponds to the faulted or unfaulted half‐unit cell. In the experiment of Figure 2, *C*_1_ was identified as faulted, while *C*_2_ was found to be unfaulted. Local atomic sites are represented as key points *P*_*i*_ (*i* = 1, 2, ⋅⋅⋅, 9): three corner holes around each half‐unit cell as *P*_1_, *P*_2_, *P*_3_, corner adatoms as *P*_4_, *P*_8_, *P*_9_, and center adatoms as *P*_5_, *P*_6_, *P*_7_.

### 2.3 Scan Module Scripts

The Scan Module contains two scripts that are essential for the room temperature experiment (thermal drift compensation and tip condition optimization), as well as a self‐driving measurement script. These scripts are used in the experiments in this manuscript. Users can add their own scripts to the scan module to customize the experiment.

The thermal drift compensation module is based on the feedforward technique presented in our previous study.^[^ ^49^ ^]^ Continuously acquired SPM images are compared using the feature point matching algorithm to output the thermal drift velocity at the minute scale, allowing correction of even non‐linear thermal drift at the day scale.

It is equipped to autonomously maintain the optimal state of the tip apex.^[^ ^38^ ^]^ Here, the CNN determines the state of the tip from the acquired images: if the CNN determines that the tip is not optimal for atomic resolution measurements, tip shaping is performed by bringing the tip close to the surface and simultaneously changing the bias current. The images are then acquired and Net1 assesses the state of the tip. These processes can be performed automatically until the tip is in a “good” state. The thermal drift module is activated during the tip optimization process to automatically compensate for thermal drift.

The self‐driving measurement module autonomously performs data acquisition based on the AI inference output and can also utilize other module functions for drift correction and tip condition optimization. As a result, our AI‐SPM system represents a departure from traditional fixed‐routine automation methods. It provides the ability for self‐driving data exploration and acquisition. A detailed description of the AI‐SPM hardware can be found in the Experimental Section.

### 2.4 Two‐Phase Training Data Acquisition

As mentioned above, even when thermal drift is compensated for at room temperature, we are facing unstable image conditions due to tip apex change. Adsorbates and defects may be present in the first place, but they may also appear during scanning, significantly influencing the SPM images. This makes us assemble a comprehensive and varied set of training data is needed. To acquire data that take into account both the quantity and variety of datasets, we have developed a “two‐phase” approach to training the AI‐SPM itself as the experiment progresses.

Phase 1 consists of collecting and creating datasets of image information obtained under different conditions. All images are collected, including those in which atoms were not clearly resolved. To obtain a large number of images categorized from *K*_0_ to *K*_10_, the tip repeatedly pokes the surface and then scans the image with a manually set drift velocity. This routine ensures diversity in the dataset regarding tip states and scanning areas. The process continues iteratively, accumulating a large dataset. This dataset is essential for training a robust model that can discriminate data quality and ensure the system's ability to autonomously acquire atomic resolution images on Si(111)‐(7 × 7).

In the second phase, the datasets of atomic resolution images is further expanded. Autonomous measurement of target atoms is performed within the self‐driving measurement module with the help of Net3, which provides information about atomic positions. One of the measurement examples is the autonomous atom manipulation technique. It uses the “atomic pen” technique^[^ ^15^ ^]^ to manipulate Si atoms within a unit cell and individually place them as single adsorptions. Repeated execution of Phase 2 can generate a larger number of adsorbate and defect patterns on the topography, thus augmenting the datasets used to train Net1, Net2, and Net3.

To improve the accuracy of Net1 and Net3, the dataset was expanded to include topography scans from different leading states and scan areas. In Phase 1 and Phase 2, 11 616 and 7269 images were acquired, respectively. Of these, 2082, 1105, and 29 145 samples and their augmented data were used to train Net1, Net2, and Net3, respectively. These data points, which are likely to be single images or instances, comprehensively cover a wide range of surface properties. The performance evaluation of Net1, Net2, and Net3 indicates they achieved accuracies of 0.93, 0.92, and 0.91, respectively (see Figure S1, Supporting Information). The accuracy of our AI models meets the criteria of sufficiently conducting real‐time AI‐driven experiments.^[^ ^35^, ^37^ ^]^

## 3 Implementation of AI‐SPM

### 3.1 Local Site Identification

**Figure 3:**
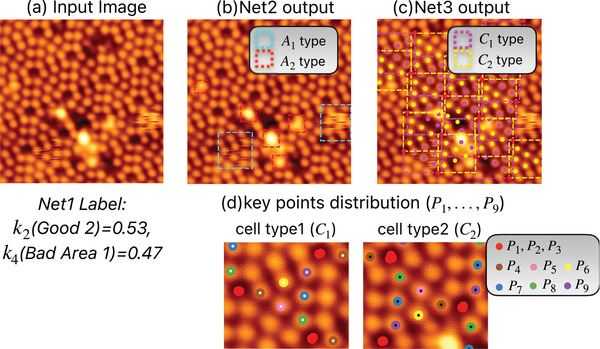
Performance of local site identification on Si(111)‐(7 × 7) surfaces. a) An image of scanning tunneling microscopy (STM) evaluated by Net1 with *k*_2_ = 0.53 and *k*_4_ = 0.47 (shown below the image), which is used for site identification as shown in (b) to (d). b) Identification of adsorbates by Net2. *A*_1_ and *A*_2_ indicate moving and stationary adsorbates, respectively. c) Classification of the half‐unit cell type by Net3. *C*_1_ and *C*_2_ are faulted and unfaulted half‐unit cells, respectively. d) Site‐specific identification of adsorbate sites and corner holes in the half‐unit cells. Each site is labeled by Net3 as *P*_*i*_(*i* = 1⋅⋅⋅9). The sample bias voltage and tunneling current in the STM measurement are *V*_*s*_=1.5 V and *I*_*t*_=200 pA, respectively.

The performance of the local site identification of the STM measurement at room temperature on Si(111)‐(7 × 7) surfaces is shown in **Figure** **3**, which shows the ability of the trained Net1, Net2, and Net3 to recognize key points of the surface for the site‐specific measurements. Images acquired as shown in Figure 3a are evaluated by Net1. In this image the (7 × 7) structure and defects and adsorbates are present. For this image, Net1 outputs weight values of *k*_2_(Good2) = 0.53 and *k*_4_(Bad area) = 0.47. This means that the tip condition is good, but surface defects and adsorbates are present. When identifying adsorbates in Net2, it can be seen in Figure 3b that it can identify stationary adsorbates (*A*_1_), surrounded by red dashed lines, and adsorbates diffusing in the half unit cell (*A*_2_), surrounded by light blue dashed lines. In Figure 3c, Net3 classifies almost all regions of the image into *C*_1_ (faulted half) and *C*_2_ (unfaulted half) and identifies individual adatoms and corner holes within these halves. Net3 can identify each adatom. As shown in Figure 3d, the individual adatoms and corner holes are represented by *P*_*i*_(*i* = 1, 2, ⋅⋅⋅, 9). The labels indicate that *i* = 1, 2, 3 corresponds to corner holes, *i* = 4, 8, 9 to corner adatoms, and *i* = 5, 6, 7 to center adatoms. These results demonstrate that our proposed Net1, Net2, and Net3 methods can identify individual atomic sites as key points at room temperature.

### 3.2 Probing for Optimal Measurement Regions

**Figure 4:**
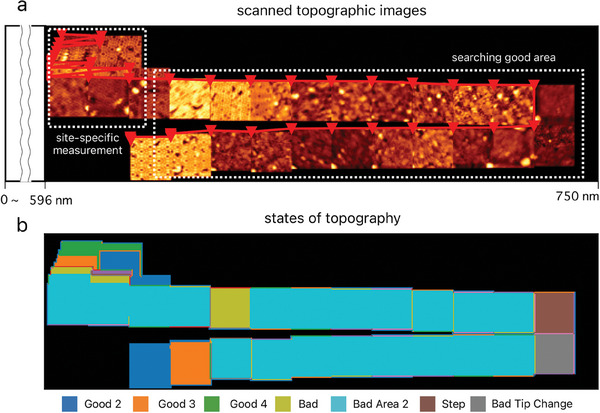
Sequential images of STM on the Si(111)‐(7 × 7) surface measured while automatically searching for the optimal measurement area and tip conditions. a) Self‐driving sequential STM imaging. The first scan starts at the upper right corner, and the scan area is automatically changed. The upper left corner of each image is indicated by an inverted triangle. The inverted triangles are connected by red lines showing the trajectory of the scan areas. b) Topmost inferred state in color of (a). All STM topographic images were acquired with 2 V sample bias, –200 pA set point, 105 s scan time, and 11.25 × 11.25 nm scan area. During this measurement, thermal drift correction is enabled by feature point matching.^[^ ^49^ ^]^

**Figure** **4** shows a sequence of consecutive room‐temperature STM images on the Si(111)‐(7 × 7) surface, in which atomic images free of defects, adsorbates, and steps are autonomously identified. In the experiment, a total of 45 consecutive images were acquired over different regions. In Figure 4a, scanning started from the upper left corner of the figure, with the upper left portion of each image marked by an inverted triangle. The trajectory of the scanned regions is shown by red lines connecting the sequence of images. Here, the scanning routine includes two critical modules to identify the atomic resolution images. First, a thermal drift correction module compensates for thermal drift to minimize image distortion.^[^ ^49^ ^]^ The second module, Net1, maintains the tip in a state conducive to atomic resolution measurements while bypassing areas affected by impurities, atomic defects, and step edges. Figure 4b shows Net1 evaluating the acquired STM images and classifying the most plausible tip or surface conditions in color.

Until now, it has been necessary to determine the appropriate measurement area and continuously monitor the condition of the tip apex. However, unlike at cryogenic temperatures where the SPM tip remains relatively stable, the tip changes frequently at room temperature. This makes it virtually impossible for researchers to conduct sustained long‐term experiments.^[^ ^16^ ^]^ As shown in Figure 4, by integrating advanced automation and deep learning techniques, we have been able to create optimal experimental conditions for exploring regions with atomic resolution. This method enables automatic identification of the appropriate tip and region at room temperature, which is essential for performing site‐specific experiments. As a result, experiments such as atomic manipulation and STS are expected to be feasible under conditions comparable to cryogenic environments.

### 3.3 Self‐Driving Scanning Tunneling Spectroscopy at Room Temperature on Different Si(111) Adatom Sites

With the methods described so far, the basic tools are now available to realize an AI‐SPM capable of self‐steering measurements. As an example of a site‐specific measurement at room temperature, we perform *I* − *V* measurements on adatoms of the Si(111)‐(7 × 7). From the obtained *I* − *V* curve, the data of dI/dV or STS as the local electronic state can be calculated. There have been previous studies of the density state of Si(111)‐(7 × 7) surfaces at both room^[^ ^50^, ^51^, ^52^, ^53^ ^]^ and low^[^ ^53^, ^54^ ^]^ temperatures. Previous studies have reported that the Si surface exhibits different electronic state behaviors depending on the temperature.

The results suggest the importance of performing the site‐specific measurement at room temperature. On the other hand, at room temperature, in addition to the thermal drift and peak instability described above, there is also the effect of thermal fluctuations in the local density of states (LDOS) itself due to the broadening of the Fermi function for the electron population, resulting in a reduction in the energy resolution of the STS measurement. Therefore, to obtain a reliable site‐specific measurement, it is necessary to acquire a large amount of data and statistically process the acquired data to address irregularities such as data variations and tip changes specific to room temperature. For site‐specific measurements at room temperature, the acquisition of large amounts of data has been very challenging with previous room‐temperature STM setups.

To ensure the acquisition of reliable data in this inherently uncertain environment, we have applied our AI‐SPM to perform a large number of *I* − *V* curve measurements on the four different adatom sites of the Si(111)‐(7 × 7) and performed statistical analysis to calculate the STS data. Specifically, it can locate individual atomic sites of the respective center and corner adatoms of each faulted and unfaulted half unit cell where adsorbates and defects were absent.

Using this approach, we obtained a total of 324 *I* − *V* curves measured at center and corner adatoms within both faulted and unfaulted unit cells. Each *I–V* curve samples 3600 points and takes 2.9 s to measure. Following these measurements, the system sequentially checks and updates the thermal drift velocity and monitors the SPM tip's condition. If Net1's detection label is not *K*_0_, *K*_1_, *K*_2_, or *K*_3_, the system initiates SPM tip conditioning. Additionally, if the detection label indicates the current area is a bad area, the system will shift to a different scanning area. The *I* − *V* data were automatically obtained in the appropriate regions and tip conditions as evaluated by AI‐SPM. This entire procedure is performed iteratively, enabling the generation of robust and reliable data under the challenging room‐temperature environments.

**Figure 5:**
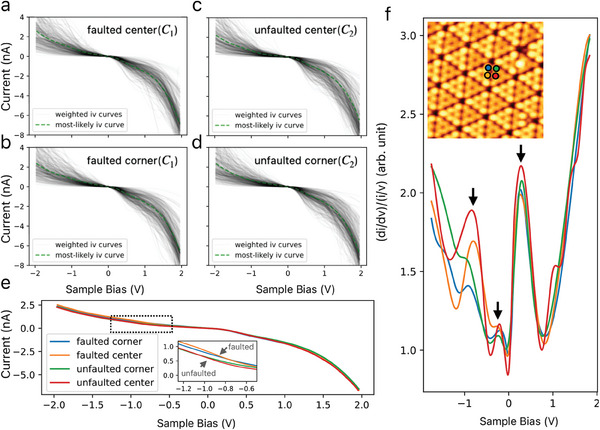
*I* − *V* and calculated curves of scanning tunneling spectroscopy (STS) on different adatom sites of the Si(111)‐(7 × 7) surface measured at room temperature. Weighted plot of *I* − *V* curves on a) faulted center adatom b) faulted corner adatom c) unfaulted center adatom d) unfaulted corner adatom. e) Averaged *I* − *V* curves of the four different adatom locations. f) STS (*dI*/*dV*) curves calculated from the four *I* − *V* curves in (e). The inset in (f) is the STM topography image acquired with the sample bias at –1.4 V, the tunneling current at 200 pA, and the scan range at 11.25 nm^2^. The four different atomic points marked by colored circles in the STM image represent the *I* − *V* measurement positions. The measurement noise is reduced by applying Savitzky–Golay and mean filters.

The obtained *I* − *V* curves are plotted individually in **Figure** **5**a–d). Our preliminary experiments have shown that the state of the tip apex changes about 6.3% of the time when the voltage is swept (see Figure S3, Supporting Information). To determine the most representative values for the *I* − *V* curves of individual atoms (Figure 5e), we used the data selection approach to obtain a group of curves with the highest trends and the mean *I* − *V* curves of the extracted group (see Experimental Section). By focusing on the region of negative sample bias, subtle differences between the curves of the faulted and unfaulted half unit cells become apparent. In this region, the conductance of the faulted unit cells is found to be greater than that of the unfaulted unit cells, supporting the higher electron density on the faulted side with a stacking fault compared to the unfaulted side.^[^ ^55^ ^]^

As shown in the inset of Figure 5f, in the STM measurements of the Si(111) surface, the contrast between the corner adatom and the center adatom in the faulted and unfaulted half‐unit cells is different in the negative sample bias region. The calculated STS results also show that under the negative sample bias region, the four site‐specific positions on the faulted/unfaulted corner and center adatoms show distinct variations (the color of the points representing the positions of the four atoms corresponds to the color of the *dI*/*dV* curves). Due to the charge transfer from the adatom to the rest atom, the center adatom, which has a larger number of neighbors to the rest atom, has a higher electron density than the corner adatom.^[^ ^52^ ^]^ This trend of the four sites is evident in our STS curves, and the positions of the peaks (downward arrow in Figure 5f) agree with the previous result.^[^ ^53^ ^]^ In addition, the most prominent peaks on the surface states of Si(111)‐(7 × 7) appear at –0.8 and 0.3 V, which is supported by the ultraviolet photoemission spectroscopy and inverse photoemission spectroscopy measurement.^[^ ^56^, ^57^ ^]^ Comparing the difference between the site‐specific STS curves, it is evident that the LDOS of the center adatom contributes most to the –0.8 V peak on the surface states. By employing statistical analysis considering the data variance in big data obtained by an AI‐assisted measurement approach to extend the I‐V curve to a datasets, our method has shown a demonstration of room temperature measurement with reliability and validity.

## 4 Discussion

Our research demonstrates that the AI‐SPM system has the potential to revolutionize room‐temperature SPM by overcoming long‐standing challenges and opening new avenues for materials characterization. This implementation can be used in both variable temperature STM and cryogenic STM, and it can be extended to other instruments in the SPM family, such as NC‐AFM. The thermal correction will be less relevant for the cryogenic environment, but the automation part still enables a completely automatic acquisition of the highest quality SPM data. Even in cryogenic environments, thermal drift can occur in experiments conducted over several days, and the tip apex can change.^[^ ^58^ ^]^ For atomic resolution experiments conducted over long periods, AI‐SPM will be a powerful tool, not limited to different types of SPM or temperature conditions.

At room temperature, various heat‐assisted processes, including diffusion, crystal growth, dislocation motion, and chemical reactions, occur at the nano‐ to atomic scale. Precise site‐specific measurements could potentially open up new areas of science if local conditions are accurately assessed. However, thermal drift and tip apex change in SPM often hinder accurate measurements. Additionally, the need to collect and statistically analyze large datasets in heat‐present environments is a significant challenge that is often beyond the capacity of manual operations. In the past, researchers have achieved excellent results with diffusion and atomic manipulation using SPM at room temperature, but it required patience, time, and luck.

To overcome these difficulties at room temperature, we have demonstrated that a deep learning‐based AI‐SPM system can be used to autonomously collect real‐time data at room temperature. A neural network is trained for comprehensive Si(111)‐(7 × 7) topography assessment, achieving an impressive accuracy of about 90% thanks to our two‐phase data acquisition scheme, which can automatically collect training datasets in real‐time measurement. Moreover, this data acquisition routine can be applied to various surfaces, allowing users to collect datasets with diversity in tip states and scanning areas, and train their own AI models for experiments.

This transformation is evident not only in its ability to assist humans in performing complex and time‐consuming operations, thus enabling automated experiments, but also in the extension of SPM to big data, aiming to unlock deeper physical discoveries as we delve into the analysis of a vast amount of data. In the future, it could be effectively used for SPM analysis at the atomic to nanometer level in materials with high temperatures and temperature variations, such as vanadium dioxide and thermoelectric materials. To achieve this vision, we need higher bandwidth automated SPM systems coupled with AI and real‐time data analysis algorithms to provide intelligent data that is directly relevant to experimental conditions and scan results. However, our current system has a limitation: it requires preliminary experiments to run the automated data acquisition routine and collect the training datasets necessary for the automated AI. In future developments, the training of large models such as Vision Transformer,^[^ ^59^ ^]^ capable of covering a wide range of material structures, would enable the use of neural networks for the analysis of general SPM data.

This manuscript has introduced the concept of a self‐driving lab, paving the way for the development of innovative approaches in atomic technology. These include the automation and optimization of manufacturing processes, and the operation, self‐replication, and self‐repair of molecular machines. These advances align closely with Drexler's anticipated integration of nanotechnology and AI.^[^ ^1^ ^]^

## 5 Conclusion

In summary, we have demonstrated that the advanced AI‐SPM system significantly improves atomic‐scale measurements by autonomously identifying atomic positions with high accuracy. The AI‐SPM's ability to detect and adopt to surface defects, manage positional drift, and cope with tip apex variations under room temperature conditions demonstrates its robustness and reliability. Successful applications on the Si(111)‐(7 × 7) surface, including defect‐free region identification and extensive current–voltage spectroscopy measurements, underscore the system's ability to enhance data acquisition for reliable material characterization. The integration of AI into SPM marks a decisive step toward more effective, precise, and reliable surface analysis at the atomic level, setting a new standard in materials characterization methods.

## 6 Experimental Section

### Sample Preparation and Experiment Environment

All experiments were performed with a home‐built STM operated at room temperature under ultrahigh vacuum conditions (< 1.0 × 10^−8^ Pa). Experiments were repeated in multiple sessions with different Pt/Ir STM tips to ensure reproducibility. An n‐type Si(111) substrate (ρ ⩽ 0.02 Ωcm) was used in this research. Atomically flat and clean Si(111)‐(7 × 7) surfaces were prepared with the standard cleaning procedure and used for data acquisition and experimental demonstration.

For data acquisition, an SPM system augmented with a deep learning model explicitly designed to facilitate autonomous measurements was built, as shown in Figure 1. The STM implementation combines an SPM instrument with a server that contains an “AI Inference” subsystem for deep learning prediction and a real‐time operating subsystem with “Scan Module” blocks that remotely control the SPM hardware (Figure 1). The control unit of the SPM instrument was built on an FPGA with remote access from a PC. The system was developed using LabVIEW, LabVIEW FPGA, and Python. The scanning and data acquisition methods were written in Python. NI PXIe‐7857r was used as the measurement board. The Scan Module block in the server contains SPM automation functions to optimize the measurement environment. It includes the scan operation with custom external scripts in Python that automated SPM measurement routines and incorporate scan functions to optimize the experimental environment. The OpenCV^[^ ^60^ ^]^ and SPMUtil^[^ ^61^ ^]^ Python packages were used for data processing and image processing. The communication between the AI inference subsystem and the SPM instrument uses a TCP protocol connection. The AI Inference subsystem runs on Python, and PyTorch^[^ ^62^ ^]^ is adopted as a machine learning framework to perform training and inference, which is accelerated by the RTX 4090 GPU for tensor computation.

### Thermal Drift Compensation

Real‐time thermal drift compensation is based on an algorithm that extracts and matches feature points across consecutive scanned images.^[^ ^49^ ^]^ By calculating the pixel shifts between successive scanned images, the scan area is adjusted to track the original region corresponding to the first image. Furthermore, the drift velocity along *x*, *y*, and *z* axes was calculated from the inter‐image shifts and the data acquisition time and used as the real‐time drift compensation velocity using a feed‐forward technique.^[^ ^19^, ^20^ ^]^ The compensation process is iterative, acquiring images and compensating for drift until the measured drift falls below a threshold of 0.2 nm. Drift compensation is configured to update the drift velocity at intervals of 10 minutes after the previous compensation is completed.

### Tip Apex Optimization

A tip apex optimization protocol is implemented to modify and evaluate tip apex conditions through controlled mechanical impacts.^[^ ^38^ ^]^ The bad tip is intentionally made to poke toward the surface, inducing apex changes. As a model case, the tip is indented 0.9 nm toward the surface relative to the 1.5 V sample bias and 200 pA tunneling current setpoint. After poking, the subsequent scanned image is input to Net1 for tip quality determination. Unsatisfactory tip conditions based on the network output triggered additional pokes. If a poke does not result in a change in the tip apex, the next poke moves the tip an additional 0.15 nm closer to the surface. In this work, the *K*_*i*_(*i* = 0, 1, 2, 3) label in Figure 2b is considered a desired tip state for further experiments. The effectiveness and reliability of this automated optimization routine at room temperature are demonstrated previously.

### Training the Deep Learning Model

The neural network types and hyperparameters for training Net1, Net2, and Net3 used in this work are listed in **Table** **1**. Net1 uses a custom convolutional network structure presented in the previous study.^[^ ^38^ ^]^ Net2 and Net3 use the YOLOv8‐small and YOLOv8‐large structures,^[^ ^63^ ^]^ and the initial trainable parameters in the network were loaded from the pre‐trained model. The YOLOv8‐large in Net3 had more trainable parameters than YOLOv8‐small, and this larger model was chosen to improve the positioning accuracy when locating the atomic key points.

**Table 1:** The detail of the neural network models.

	Model type			Hyperparameter		
Name	Architecture	Task	Output type	Learning rate	Training epoch	Batch size
Net1	Custom CNN	tip and sample classification	scalar	5 × 10^−4^	720	16
Net2	YOLOv8s	adsorption detection	bounding boxes	2 × 10^−3^	150	16
Net3	YOLOv8l	atomic site detection	bounding boxes/key points	1 × 10^−3^	300	16

In the first stage of training Net1, Net2, and Net3, the input images were resized to 256 × 256 pixels. The training dataset could be automatically labeled by the trained model. The labeled dataset was then exported to CVAT for manual validation. Among more than 10 000 images from experiments, the data were selected with different appearances and balanced the number of each category to build the training dataset. Within this dataset, 2082 samples were designated for training Net1, 1105 samples from 255 images for training Net2, and 29 145 samples from 545 images for training Net3. These data sets were divided into 80% for training and 20% for validation. Then, data augmentation,^[^ ^64^ ^]^ which included random affine transform, image cropping, and contrast change, was applied separately to the training and validation datasets, expanding the datasetsby a factor of 5. The three networks (Net1, Net2, and Net3) were trained using the AdamW^[^ ^65^ ^]^ optimizer.

### *I* − *V* Curves Similarity Metric and Selection

To extract the high consistency tendens from a group of *I* − *V* curves, the cosine similarity metric ϕ is used for statistical analysis. The similarity ϕ of two *I* − *V* curves *I*_*i*_, *I*_*j*_ (*i*, *j* = 1, 2, ⋅⋅⋅, *m*) each containing *n* count data points in one curve is calculated by the following equation.

(1) $$\phi \left(I_{i},I_{j}\right)=\frac{\Sigma_{k=1}^{n}I_{i}\left[k\right]\cdot I_{j}\left[k\right]}{\sqrt{\Sigma_{k=1}^{n}I_{i}{\left[k\right]}^{2}\cdot \Sigma_{k=1}^{n}I_{j}{\left[k\right]}^{2}}}$$

In particular, ϕ(*I*_1_, *I*_2_) ranges from –1 to 1, where ϕ(*I*_1_, *I*_2_) = 1 indicates that the two curves are identical. By calculating ϕ among all *I* − *V* curves, a group of curves could be obtained that can represent the most common data of the whole data. The main group selection method can be applied in two ways, based on the whole data (overall selection) or based on selected data (reference data‐based selection). As for the overall selection method, given the whole *I* − *V* curve data as *I*_1_, *I*_2_, ⋅⋅⋅, *I*_*l*_, ⋅⋅⋅, *I*_*m*_, the mean value of the cosine similarity (ϕ_mean_[*I*_*l*_]) was calculated for each *I* − *V* curve relative to all other curves.

(2) $$\phi_{\mathrm{mean}}\left(I_{l}\right)=\sum_{m}^{i}\frac{\phi (I_{l},I_{i})}{m}$$

The ϕ_mean_([*I*_*l*_]) for *I*_*l*_ represents the common property for all *I*−*V* curves, so a threshold *T*_1_ is then applied with the condition ϕ_mean_([*I*_*l*_]) > *T*_1_ to select all *I*_*l*_ curves that satisfy this condition as a main group. The reference data‐based selection method involves selecting a group of data similar to the reference data, which is one of the *I* − *V* curves from the *I* − *V* curve group, as the reference data *I*_ref_. For an *I* − *V* curve *I*_*l*_, a threshold *T*_2_ is specified and the condition ϕ(*I*_ref_, *I*_*l*_) > *T*_2_ is used to determine whether *I*_*l*_ can be added to the main group based on *I*_ref_. Before comparing the relationship between the *I* − *V* curves, the measurement noise is reduced using Savitzky–Golay^[^ ^66^ ^]^ and mean filters. After the main group is extracted, the most suitable tendency in the group could be extracted by taking the average value in the *I* − *V* curves and smoothing with a 1D spline function.

### Statistical Analysis

This study preprocessed all SPM image data using plane fit subtraction and Gaussian–Hann filters. For the *I–V* curve data, Savitzky–Golay^[^ ^66^ ^]^ and mean filters were applied, followed by smoothing with a 1D spline function to minimize noise. The data processing and analysis methods proposed in this paper are implemented in Python. Scripts for all methods are available as open‐source resources.^[^ ^67^ ^]^

## Conflict of Interest

The authors declare no conflict of interest.

## Supporting information

Supporting Information

## Data Availability

The data that support the findings of this study are openly available in compositenet for spm at https://github.com/DIAOZHUO/composite_net_for_spm, reference number 68.
