# Supplementary material for: AI‐Equipped Scanning Probe Microscopy for Autonomous Site‐Specific Atomic‐Level Characterization at Room Temperature
Source: Small Methods. 2024 Sep 6;9(1):2400813. doi: 10.1002/smtd.202400813 (PMC11740938; doi:10.1002/smtd.202400813)
Supplement: Supplementary file 1 — Supporting Information [file SMTD-9-2400813-s001.pdf]

small
methods

Supporting Information

for *Small Methods*, DOI 10.1002/smtd.202400813

AI-Equipped Scanning Probe Microscopy for Autonomous Site-Specific Atomic-Level Characterization at Room Temperature

Zhuo Diao, Keiichi Ueda, Linfeng Hou, Fengxuan Li, Hayato Yamashita and Masayuki Abe**

Supporting Information of AI-equipped scanning probe microscopy for autonomous site-specific atomic-level characterization at room temperature

Zhuo Diao,[*,†] Keiichi Ueda,[†,‡] Linfeng Hou,[†] Fengxuan Li,[†] Hayato Yamashita,[†] and Masayuki Abe[*,†]

†*Graduate School of Engineering Science, Osaka University, 1-3 Machikaneyama-Cho, Toyonaka, Osaka 560-8531, Japan*

‡*Tokyo Metropolitan Industrial Technology, Research Institute, 2-4-10 Aomi, Koto-Ku, Tokyo, 135-0064, Japan*

E-mail: diao.zhuo.es@osaka-u.ac.jp; abe.masayuki.es@osaka-u.ac.jp

Supporting Information Available

Model evaluation

In the experiments of our artificial intelligences scanning probe microscopy (AI-SPM), several metrics are introduced to evaluate model performance, specifically to validate classification and detection accuracy. For Net1, a confusion matrix is used to verify classification accuracy, as shown in Figure S1(a). In the matrix, each element represents the number of actual instances (horizontal axis) and the Net1 predictions (vertical axis), normalized by the number

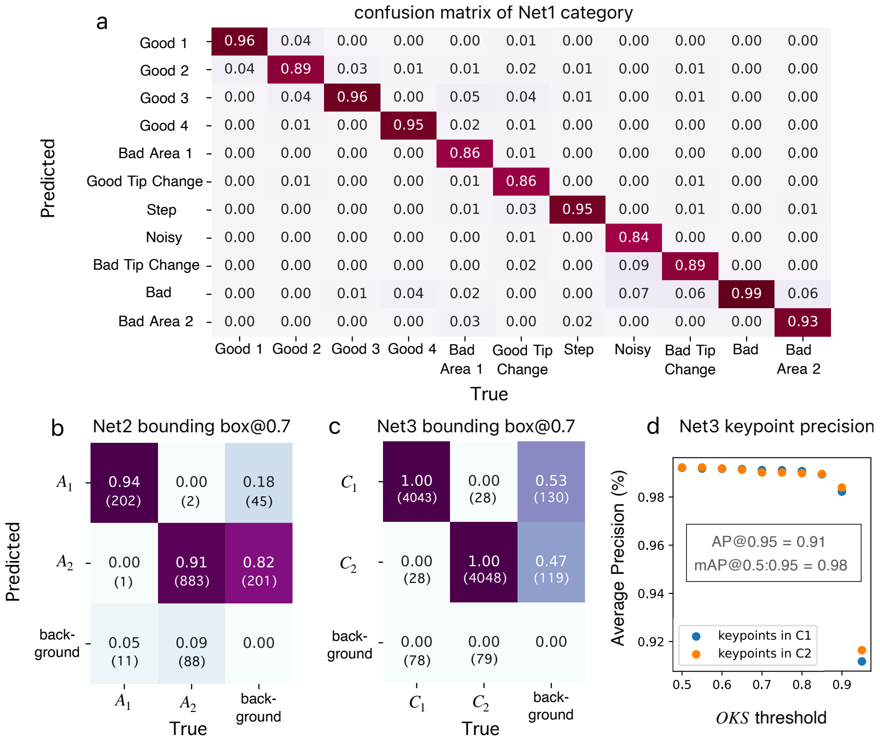

Figure S1: Performance of the artificial intelligences (AIs) equipped in our scanning probe microscopy (AI-SPM). (a) to (c) Confusion matrix showing the classification accuracy of Net1 to Net3, respectively. In (b) and (c), the background means that the AI does not treat the detection object as an interest to accurately identify, and a value of Intersection Over Union ($IoU \geq 0.7$) is defined as the true positive case. The numbers in brackets within the matrix elements represent the number of test samples. (d) Object Keypoint Similarity (OKS) threshold dependence of the Average Precision (AP) analysis of Net3 for atomic point detection. Mean AP (mAP) value is calculated corresponding to OKS from 0.5 to 0.95.

of ground truth instances for each class. The diagonal elements represent the *Recall* metric, defined as

$$Recall = \frac{TP}{TP + FN}, \tag{1}$$

where True Positive (TP) is the number of predictions correctly identified as positive among the actual positives, and False Negative (FN) is the number of actual positives incorrectly predicted as negative. The recall metric serves as an indicator of reproducibility when the model is applied within a measurement system. For example, as shown in the first row

and column of Fig. S1(a), scanning with a tip in the "Good 1" state provided the system with a 0.98 probability of successful identification. The average *Recall* across all classes was 0.93, and the *Recall* for the binary judgment of whether a tip was good or bad was 0.98. Compared to our previous study,[1] which used the same algorithm of Net1, we confirmed the improvement of the *Recall* value of Net1 from 87% to 93%. This improvement is attributed to the expansion of the datasets to 2000 samples.

For Net2, the performance of moving adsorption (A_1) and non-moving adsorption (A_2) detection is validated. To define the success or failure of the detection, the Intersection Over Union (IoU) metric, which quantifies the overlap between the predicted bounding box and the ground truth bounding box for a given object instance, is introduced. It is defined as the intersection area between the predicted box and the ground truth box divided by the union of the two boxes,

$$IoU = \frac{S_{\text{overlap}}}{S_{\text{union}}}, \tag{2}$$

where S_{overlap} is the overlap area of the predicted and ground truth boxes, and S_{union} is their union area covered by both boxes. We defined a threshold of $IoU = 0.7$ to determine whether a prediction should be considered a true or false positive. Specifically, a predicted box with $IoU \geq 0.7$ with respect to the ground truth is counted as a true positive (TP), while a false negative (FN) indicates a failure to detect a ground truth object. Using this categorization, a confusion matrix can then be constructed to summarize the performance of each object class on the validation datasets. Figure. S1 (b) shows the normalized confusion matrix when $IoU \geq 0.7$ was used as the threshold for dividing the predictions in the validation datasets. The category label also includes "background", which represents the object that does not contain a target. The reason why a certain amount of background is misidentified as a target object may be that our detection model repeatedly detects certain targets. The average recall for detecting A_1 and A_2 is 0.92.

For Net3, Object Keypoint Similarity (OKS) has been introduced to verify the accuracy

of bounding box (C_1, C_2) and key point (P_i) detection,

$$OKS = \frac{\Sigma_i^n \exp(-d_i^2/2s^2)\delta(v_i > 0)}{\Sigma_i^n \delta(v_i > 0)}, \tag{3}$$

where n is the number of key points, d_i is the Euclidean distance between the predicted key point and its corresponding ground truth, s is the region scale of the detected object, and $\delta(v_i > 0)$ is an indicator function that resolves to 1 if the key point is visible or 0 if it is occluded. OKS ranges from 0 to 1, where 1 indicates perfect alignment of the predicted key points with the ground truth. A threshold of $OKS = 0.5$ has been used to determine true versus false predicted key points, and $OKS \geq 0.95$ can provide a rigorous evaluation precision of the atom localization quality. The overall key point detection performance can then be quantified by the proportion of key points with OKS above a threshold. This is called the mean Average Precision (mAP) for one of the key point classes.

Figure. S1 (c) shows the normalized confusion matrix of two unit cells (C1, C2) and background. Here, similar to Net2, $IoU = 0.7$ was chosen as the threshold to plot the normalized confusion matrix of two unit cells (C1, C2) and background. A significant amount of unit cell training samples allowed the *Recall* value to approach its maximum performance level of 1.0.

In addition, the ability to accurately locate atomic key points can be evaluated by the OKS value. By choosing OKS thresholds varying by 0.05 from 0.5 to 0.95, the respect of the average precision detected in C_1 and C_2 are plotted in Figure S1 (d). mAP was above 0.98, and as the most stringent metric, the mAP value at $OKS \geq 0.95$ was 0.91. Overall, these models are trained on the diversity of the dataset acquired in real time by a step-up data acquisition scheme. All models achieved high accuracy performance even for real-time data at room temperature, ensuring robustness of measurements and localization performance for SPM automation.

Statistical methods in room temperature $I - V$ measurements

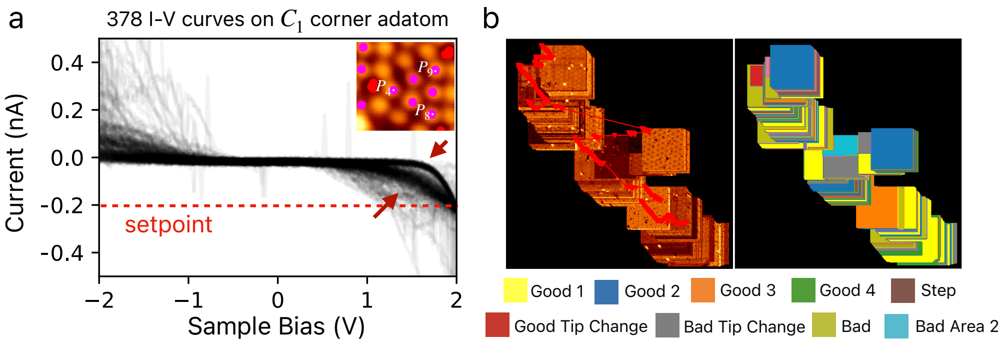

Figure S2: Site-specific $I - V$ measurement by AI-SPM on the Si(111) − (7×7) surface at room temperature. A tip of scanning tunneling microscopy (STM) was repaired while searching the region, and data were automatically acquired in the region deemed appropriate for the $I - V$ measurement. (a) $I - V$ curves measured at the corner adatoms of faulted half-unit cells (C_1) on the Si(111) − (7×7) surface. A total of 378 curves are superimposed. The horizontal red dashed line indicates the set point of the tunneling current before the start of the IV scan. "P_4, P_8, and P_9 in the inset are corner adatoms within C_1 identified by Net3 where the $I - V$ curves were automatically measured. In reality, measurements were taken at four different atomic sites on Si(111). (b) Topmost inferred state of the STM images during the self-driving $I - V$ measurements by our AI-SPM. The notation is the same as in Figure. 4(b) of the main manuscript.

Figure. S2 shows the results of a long-term site-specific $I - V$ measurement of scanning tunneling microscopy (STM) performed to verify the rate of change of the probe. The measurement is the same as in Figure 5 in the main text, but a different STM tip was used. The measurement took 58 hours and a total of 695 topographic STM images and 2832 $I - V$ curves were obtained. The selected 378 $I - V$ curves acquired at the corner adatom of the half unit cell (the 4th, 8th and 9th points detected by Net3) are shown in Figure. S2(a). The trajectory of the imaging area and the top inferred state of the STM images during the self-driving $I - V$ measurements by our AI-SPM are shown in Figure. S2(b). The $I - V$ curve measurement includes both a forward sweep (I_{fw}) from 2 V to −2 V of sample bias and a backward sweep (I_{bw}) from −2 V to −2 V of sample bias. The scan is initiated from the target atom where the z position is specifically at a −200 pA setpoint at a 2 V

sample bias. The $I-V$ curves in Figure. S3(a) include both I_{fw} and I_{bw}, and almost all of the curves align precisely at the -200 pA setpoint under a 2 V sample bias position. This observation indicates that drift along the x, y, and z axes has been effectively corrected, providing compelling evidence that the system can mitigate the effects of thermal drift even during room temperature measurements.

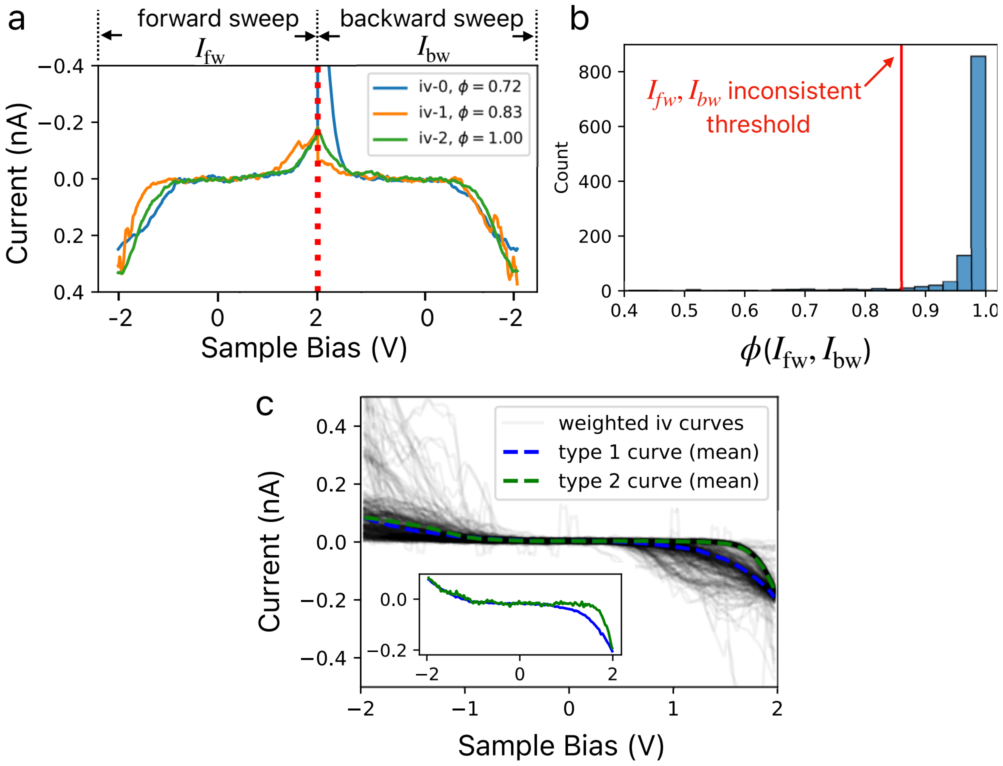

Figure S3: Statistical analysis of 378 curves on the C_1 corner atom. (a) Three raw data("iv-0", "iv-1", "iv-2") of the IV measurement taken by setting the sample bias from 2 V to -2 V as the forward sweep and then -2 V to 2 V as the backward sweep, starting from -200 pA set point at 2 V sample bias. (b) Histogram of the similarity metric $\phi(I_{\mathrm{fw}}, I_{\mathrm{bw}})$ between the forward $I-V$ curve I_{fw} and the backward $I-V$ curve I_{bw}. (c) The result of two $I-V$ curves representing the mean of the two trend groups. The type 1 curve is selected by the total data-based selection method, and the type 2 curve is selected by the reference curve-based selection method (see Methods of the main manuscript).

However, due to various environmental changes during the measurement process, the numerical values of I_{fw} and I_{bw} may not always match. In cases where the tip stabilizes and the surface remains undisturbed, I_{fw} and I_{bw} tend to agree. To assess the degree of agreement between the I_{fw} and I_{bw} curves, we used the cosine similarity metric (see Method).

In Fig. S3(a), three curves (iv-0, iv-1, and iv-2) are shown as examples. As in the case of iv-0 and iv-1, ϕ values of 0.72 and 0.83, respectively, indicate that a change in tip apex has occurred. In contrast, when there is a high degree of consistency, as observed in the curve of "iv-2," the curve reaches a ϕ value of 1.00.

All $\phi(I_{\rm fw}, I_{\rm bw})$ values across a set of $I - V$ curves are calculated and plotted in the histogram in Figure. S3(b). We defined $\phi(I_{\rm fw}, I_{\rm bw}) < 0.86$ as an indication of an error in $I - V$ measurements caused by changes in measurement conditions, and the probability of encountering such measurement discrepancies using our STS parameter was 6.3%. The reason for these discrepancies is often related to the effect of the applied sample bias on the tip apex, which can be likened to a probe conditioning process.[2] These cases are often observed during measurements, manifesting as tip changes or impurities dropping on the surface, and are largely unavoidable when operating at room temperature.

Despite the potential influence of an unstable measurement environment as described earlier, trends in surface properties can still be observed, especially when supported by adequate data. Considering that all curves in the plot have been weighted, the areas with denser line colors indicate regions of higher weight, reflecting a more pronounced tendency in the $I - V$ curve within those specific regions. Within the range of 1 to 2 V sample bias, two $I - V$ curves show the most prominent directions. To distinguish and represent these two distinct trends, an algorithm based on cosine similarity (see Methods) is used to statistically differentiate and select the sets of $I - V$ curves that represent these two trends. The mean values of all curves within each set corresponding to the two trends are plotted as blue and green dotted lines in Figure. S3(c). The reason for the differentiation of these two trends on C_1 type corner adatoms, despite their identical nature, could be attributed to variations in the density of the state of the STM tip apex during the $I - V$ curve measurements.

STS result variation in Si(111)-(7×7) four-site adatom

Curves of scanning tunneling spectroscopy (STS) is calculated using $I - V$ curves as shown in Figure S4 .

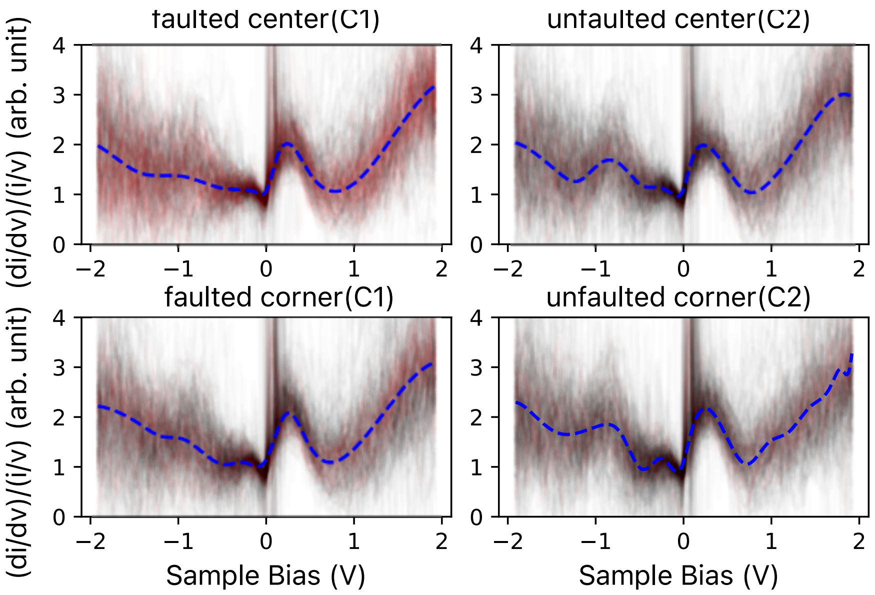

Figure S4: Weighted plots of scanning tunneling spectroscopy (STS) at (a) faulted center adatom, (b) faulted corner adatom, (c) unfaulted center adatom, and (d) unfaulted corner adatom, respectively. All the curves are calculated from the $I-V$ curve data shown in Figure 5(a)-(d). The "$I-V$ curves similarity metric and selection" method is used to determine the appropriate I-V curves in the data with variation. The STS curves in the selection group are plotted in red, while those not in the selection group are plotted in black. The blue dashed line represents the mean value of the selection group, which corresponds to the same data as the STS results shown in Figure 5(f).

References

(1) Diao, Z.; Hou, L.; Abe, M. Probe conditioning via convolution neural network for scanning probe microscopy automation. *Applied Physics Express* **2023**, *16*, 085002.

(2) Wang, S.; Zhu, J.; Blackwell, R.; Fischer, F. R. Automated Tip Conditioning for Scanning Tunneling Spectroscopy. *The Journal of Physical Chemistry A* **2021**, *125*, 1384–1390, PMID: 33560124.
